# Axonal Tract Reconstruction Using a Tissue-Engineered Nigrostriatal Pathway in a Rat Model of Parkinson’s Disease

**DOI:** 10.3390/ijms232213985

**Published:** 2022-11-12

**Authors:** Laura A. Struzyna, Kevin D. Browne, Justin C. Burrell, Wisberty J. Gordián Vélez, Kathryn L. Wofford, Hilton M. Kaplan, N. Sanjeeva Murthy, H. Isaac Chen, John E. Duda, Rodrigo A. España, D. Kacy Cullen

**Affiliations:** 1 Center for Brain Injury & Repair, Department of Neurosurgery, Perelman School of Medicine, University of Pennsylvania, Philadelphia, PA 19104, USA; 2 Center for Neurotrauma, Neurodegeneration & Restoration, Michael J. Crescenz Veterans Affairs Medical Center, Philadelphia, PA 19104, USA; 3 Department of Bioengineering, School of Engineering and Applied Science, University of Pennsylvania, Philadelphia, PA 19104, USA; 4 New Jersey Center for Biomaterials, Rutgers University, Piscataway, NJ 08854, USA; 5 Department of Neurology, Perelman School of Medicine, University of Pennsylvania, Philadelphia, PA 19104, USA; 6 Department of Neurobiology & Anatomy, College of Medicine, Drexel University, Philadelphia, PA 19129, USA

**Keywords:** regenerative medicine, Parkinson’s disease, nigrostriatal pathway, dopamine, neurons, tissue engineering

## Abstract

Parkinson’s disease (PD) affects 1–2% of people over 65, causing significant morbidity across a progressive disease course. The classic PD motor deficits are caused by the degeneration of dopaminergic neurons in the substantia nigra pars compacta (SNpc), resulting in the loss of their long-distance axonal projections that modulate striatal output. While contemporary treatments temporarily alleviate symptoms of this disconnection, there is no approach able to replace the nigrostriatal pathway. We applied microtissue engineering techniques to create a living, implantable tissue-engineered nigrostriatal pathway (TE-NSP) that mimics the architecture and function of the native pathway. TE-NSPs comprise a discrete population of dopaminergic neurons extending long, bundled axonal tracts within the lumen of hydrogel micro-columns. Neurons were isolated from the ventral mesencephalon of transgenic rats selectively expressing the green fluorescent protein in dopaminergic neurons with subsequent fluorescent-activated cell sorting to enrich a population to 60% purity. The lumen extracellular matrix and growth factors were varied to optimize cytoarchitecture and neurite length, while immunocytochemistry and fast-scan cyclic voltammetry (FSCV) revealed that TE-NSP axons released dopamine and integrated with striatal neurons in vitro. Finally, TE-NSPs were implanted to span the nigrostriatal pathway in a rat PD model with a unilateral 6-hydroxydopamine SNpc lesion. Immunohistochemistry and FSCV established that transplanted TE-NSPs survived, maintained their axonal tract projections, extended dopaminergic neurites into host tissue, and released dopamine in the striatum. This work showed proof of concept that TE-NSPs can reconstruct the nigrostriatal pathway, providing motivation for future studies evaluating potential functional benefits and long-term durability of this strategy. This pathway reconstruction strategy may ultimately replace lost neuroarchitecture and alleviate the cause of motor symptoms for PD patients.

## 1. Introduction

Parkinson’s disease (PD) is a progressive neurodegenerative disease that results in substantial morbidity across a protracted and worsening disease course [1,2,3]. PD affects 1–2% of people over the age of 65, and the typical motor symptoms of PD are caused by selective degeneration of dopaminergic neurons in the substantia nigra pars compacta (SNpc) and loss of their long-distance axonal projections into the striatum. This deafferentation results in the loss of finely tuned dopamine delivery to the striatum and therefore disrupts the function of key motor control circuits. Although many PD treatments have been developed, most strategies focus on alleviating the symptoms resulting from the stereotypical neuronal degeneration rather than addressing the underlying pathology. Such treatments include the use of dopamine-replacement therapies (e.g., 3,4-dihydroxy-L-phenylalanine (L-DOPA) and dopamine agonists), which aim to correct the underproduction of dopamine caused by neuron/axon loss [2]. While these dopamine-related therapies have assuaged symptoms to a certain degree, side effects such as dyskinesias and motor fluctuations often develop and treatment efficacy declines over time [4]. Alternatively, deep brain stimulation (DBS) systems can be implanted via stereotactic neurosurgical techniques to alleviate motor symptoms by modulating basal ganglia network function. While DBS has also provided symptom improvements, surgical procedures, even minimally invasive ones, have inherent risks, and the stimulation can negatively affect other functions including gait, cognition, and speech [5]. As with pharmaceutical therapies, DBS efficacy often declines over time as the state of the disease progresses. While neuroprotective treatments would be ideal to prevent ongoing degeneration, so far, trials utilizing this approach have only marginally slowed disease progression [6]. Furthermore, as ≥60% of SNpc neurons and their axonal projections to the striatum have already degenerated by the time of motor symptom onset, even an effective neuroprotective strategy would be unable to correct for the substantial neuron/axon loss that would have occurred prior to treatment with currently available diagnostic abilities [7].

These gaps in treatment have spawned significant efforts toward replacing components of the nigrostriatal pathway via (1) tissue grafts, (2) cell implantation, and (3) scaffold-based methods. Clinical trials have revealed positive functional improvements from tissue grafts when a population of at least 80,000 transplanted dopaminergic neurons is maintained in the striatum [8]. However, while grafts or cells transplanted ectopically into the striatum are capable of providing living “factories” for dopamine release, such strategies do not recreate the nigrostriatal pathway [9]. As such, normal feedback circuitry would not govern the activity of these implanted cells, and thus, striatal neurons would likely not receive correctly regulated dopamine levels [10,11]. In fact, this has led to a clinical syndrome of “runaway dyskinesia” in some patients in which unregulated dopamine release causes dyskinesia that is difficult, if not impossible, to resolve [12]. On the other hand, studies involving cell implantation into the SNpc with and without other strategies to provide axon guidance have shown axonal outgrowth to the striatum, although this outgrowth was oftentimes insufficient and non-specific and it is doubtful it could be replicated in the much larger human brain [13]. In addition, widespread cell loss, nonspecific cell type differentiation, and neoplastic transformation have limited the success of stem cell implantations. Lastly, although biomaterial scaffolds have assisted axonal regrowth in the central nervous system (CNS), biomaterial treatments alone do not address the loss of SNpc neurons and underlying circuitry that is the root of the motor symptoms [14,15,16].

To address these challenges, we have developed tissue-engineered nigrostriatal pathways (TE-NSPs) as a strategy to physically reconstruct the nigrostriatal pathway. TE-NSPs are designed to replace both the lost dopaminergic neurons in the SNpc and their axonal projections to the striatum—thereby allowing implanted cells/tissue to be subject to the typical synaptic regulation that governs dopaminergic SNpc neuron activity and thus “close the loop” by reestablishing this critical circuit for motor control feedback. TE-NSPs are anatomically inspired three-dimensional (3D) cylindrical constructs comprising a discrete dopaminergic neuron population projecting long, bundled axonal tracts [17,18,19,20,21,22]. These neuronal–axonal constructs are encapsulated within a miniature tubular hydrogel featuring an interior extracellular matrix (ECM) core that supports the neurite outgrowth in vitro. Their miniature form factor (only several hundred microns in diameter) allows for minimally invasive delivery into the brain [18], while the biomaterial encasement protects their preformed cytoarchitecture [22]. We previously reported the construction of transplantable constructs that mimic the general cytoarchitecture of the nigrostriatal pathway, while providing the first evidence of dopaminergic axonal extension >6 mm [19]. These constructs were generated using embryonic rat midbrain preparations, which contained a relatively low percentage of dopaminergic neurons (<10%) [19].

In the current work, we advanced TE-NSP technology to generate constructs with higher dopaminergic purity by isolating cells from the ventral mesencephalon of transgenic rats expressing green fluorescent protein (GFP) in dopaminergic neurons and performing fluorescent-activated cell sorting (FACS). We then optimized biomaterial and media conditions to maximize the health and outgrowth of these enriched TE-NSPs. We also evaluated TE-NSPs containing a population of striatal neurons at the opposite end to measure structural and functional integration with a surrogate end target in vitro. Finally, we assessed the survival and efficacy of TE-NSPs implanted to span the nigrostriatal pathway in an athymic rat model of PD generated using a unilateral 6-hydroxydopamine (6-OHDA) lesion of the SNpc. Collectively, these studies advance the biofabrication, characterization, and functional validation of implantable anatomically inspired microtissue as a novel pathway reconstruction strategy for the treatment of PD.

## 2. Results

### 2.1. Optimization of TE-NSPs Fabricated with Enriched Dopaminergic Population

**Figure 1:**
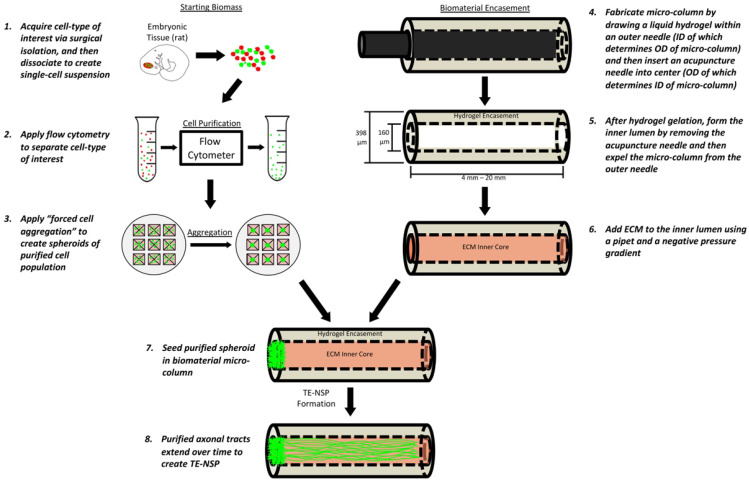
Fabrication of TE-NSPs. In order to create the cell aggregates, cells are first obtained from the ventral mesencephalon of transgenic rat embryos. In order to enrich the cell population for dopaminergic neurons, the cells are dissociated and run through a flow cytometer where fluorescent labels can be used to perform FACS. The purified cells are then centrifuged in custom, inverted PDMS wells to create cell aggregates. In order to generate the biomaterial micro-columns, agarose is gelled in a capillary tube containing a needle. Once the agarose has hardened, the needle is removed to create a hollow agarose tube, which is then filled with an ECM core. Lastly, the aggregates are inserted into the ends of the tubes, where they extend neurites over days to weeks in vitro.

We initially fabricated TE-NSPs utilizing cell aggregates generated with primary embryonic midbrain neurons. However, as the primary midbrain neuron preparation yielded a relatively low percentage of dopaminergic neurons (<10%), we used a transgenic rat model in combination with FACS to produce an enriched dopaminergic population. Here, transgenic male rats expressing GFP in their dopaminergic neurons (NTac:SD-Tg(TH-EGFP)24Xen; Taconic model #12141-M) were bred with wild-type females. At embryonic day 14, the standard midbrain neuron preparation was performed, followed by FACS to selectively isolate the GFP+ dopaminergic neurons (Figure 1).

**Figure 2:**
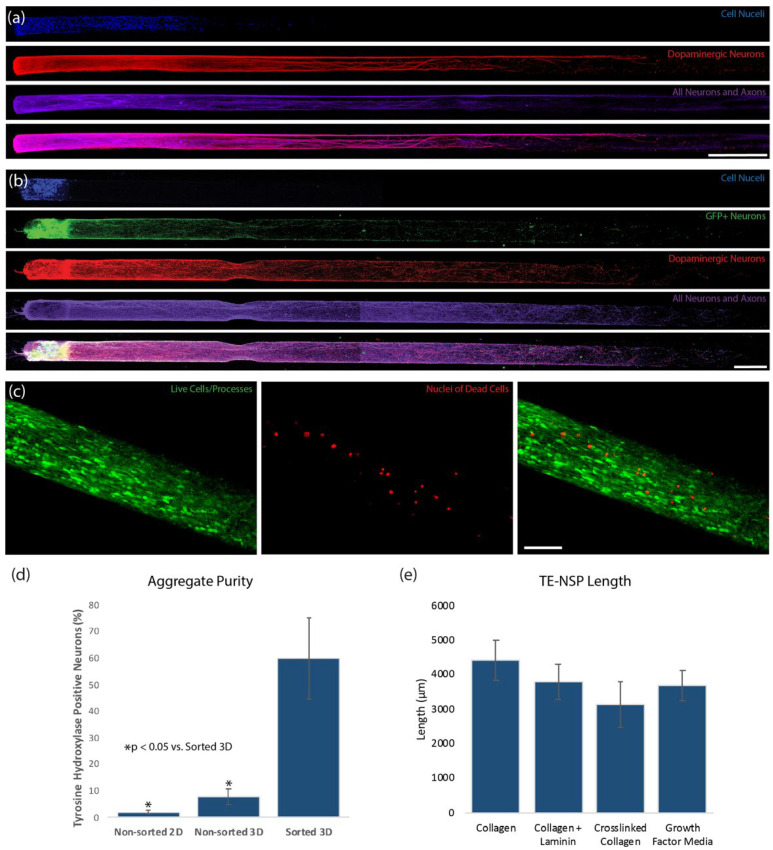
Optimization of Enriched TE-NSPs. (**a**,**b**) Confocal reconstructions of representative constructs at 14 DIV labeled via immunocytochemistry to denote all neurons/axons (β-tubulin III, purple) and dopaminergic neurons/axons (TH, red), with nuclear counterstain (Hoechst, blue). Both constructs demonstrated the desired cytoarchitecture consisting of discrete cell bodies and axonal regions. (**a**) A construct generated with neurons isolated from the ventral mesencephalon absent in any enrichment process. (**b**) A construct containing a neuronal population that was enriched for GFP+ dopaminergic neurons using FACS. (**c**) Viability assay on enriched TE-NSPs showing live cells/processes (green) and the nuclei of dead cells (red). (**d**) TE-NSPs generated using FACS-enriched aggregates (“Sorted 3D”) demonstrated a higher percentage of dopaminergic neurons in comparison to TE-NSPs plated with aggregates (“Non-sorted 3D”) or planar cultures (“Non-sorted 2D”) made from the standard midbrain isolation at 14 DIV (n = 5 TE-NSPs each group; Mann–Whitney test, *p* < 0.05). (**e**) One-way ANOVA (*p* = 0.3297) determined that all ECM cores and high growth factor media resulted in statistically equivalent TE-NSP length at 14 DIV (n = 6 TE-NSPs per group). Data presented as mean ± standard deviation. Scale bar (**a**) = 500 µm. Scale bar (**b**) = 250 µm.

Utilizing the forced neuronal aggregation technique that we previously optimized with the standard population [19], we were able to fabricate TE-NSPs displaying the desired cytoarchitecture with the enriched population (Figure 2b). These TE-NSPs extended robust processes along the length of the micro-column and remained healthy for at least several weeks in culture. Indeed, a LIVE/DEAD assay revealed an average cell viability of 85.2 ± 6.1% (mean ± standard deviation) out to 14 days in vitro (DIV) (Figure 2c). Importantly, this protocol yielded a neuronal population containing an average of approximately 60% dopaminergic neurons (Figure 2d).

We next tested the effects of various ECM constituents, as well as growth factors, on the axonal outgrowth of the enriched TE-NSPs. While the use of 1 mg/mL rat tail collagen as the inner core produced TE-NSPs with the longest axonal outgrowth at 14 DIV, these results did not differ statistically from those found when laminin was added or when crosslinked collagen was used (Figure 2e). Likewise, the use of media containing high concentrations of growth factors did not result in enhanced axonal outgrowth (Figure 2e). As the majority of the enriched constructs did not measure 5 mm (the length of the nigrostriatal pathway in rats) by 14 DIV, we monitored their growth to 21 DIV and found that the average length of the constructs was 4590 ± 1111 μm (mean ± standard deviation). Thus, by 21 DIV, many of the enriched TE-NSPs achieved the 5 mm length necessary to span the nigrostriatal pathway in rats. 

### 2.2. Dopaminergic Neuron Machinery for Dopamine Uptake and Release

**Figure 3:**
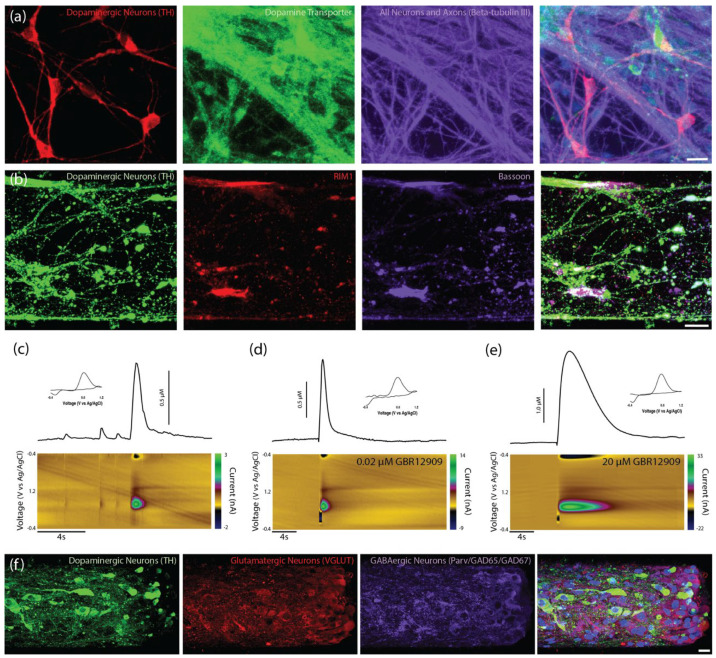
Characterization of unidirectional TE-NSPs. (**a**) Representative confocal reconstruction at 14 DIV of a standard midbrain population containing dopaminergic neurons labeled via immunocytochemistry to denote dopaminergic neurons/axons (TH, red), the dopamine transporter protein (dopamine transporter, green), and all neurons/axons (β-tubulin III, purple). Dopaminergic neurons exhibit expression of the dopamine transporter (DAT), a protein involved in dopamine recycling. (**b**) Representative confocal reconstruction at 14 DIV of an enriched TE-NSP labeled via immunocytochemistry to denote dopaminergic neurons/axons (TH, green), RIM proteins (RIM1, red), and bassoon proteins (Bassoon, purple). The RIM and bassoon proteins colocalize on putative dopaminergic varicosities. (**c**–**e**) FSCV was used to measure dopamine release from TENSPs: A stimulating electrode was positioned to span the dopaminergic aggregate while a carbon-fiber recording electrode was placed within the aggregate. Representative color plots displaying the current recorded as the potential of the carbon fiber electrode were linearly scanned from −0.4 to 1.2 V and back to −0.4 V vs. Ag/AgCl every 100 ms (bottom). Cross-sections from the color plots display individual cyclic voltammograms exhibiting an oxidation peak at 0.65 V and a reduction trough at −0.3 V, which are characteristic of dopamine (inset). Representative time vs. concentration plots of evoked dopamine release (top). (**c**) Evoked dopamine release was recorded from unidirectional enriched dopaminergic neurons in TE-NSPs. (**d**,**e**) Evoked dopamine release was also recorded from unidirectional enriched TE-NSPs following perfusion with different concentrations of the DAT inhibitor GBR12909. The concentration of GBR12909 in (**d**) was 0.02 μM and in (**e**) was 20 μM. The application of the DAT inhibitor GBR12909 increased both the maximum concentration of evoked dopamine and the duration of dopamine release. (**f**) Confocal reconstructions of FACS enriched aggregates at 35 DIV labeled via immunocytochemistry to denote all dopaminergic neurons/axons (TH, green), glutamatergic neurons (VGLUT, red), and GABAergic neurons (Parvalbumin + GAD65 + GAD67, purple), with nuclear counterstain (Hoechst, blue). Following maturation over weeks in vitro, sorted aggregates demonstrated the presence of dopaminergic, GABAergic, and glutamatergic neurons. Scale bar (**a**) = 20 µm. Scale bar (**b**) = 25 µm. Scale Bar (**f**) = 20 µm.

Immunocytochemistry was used to label dopaminergic neurons for markers of elements involved in dopamine uptake and release. The dopamine transporter (DAT) is expressed heavily along dopaminergic neuron cell bodies and neurites and plays a large role in recycling dopamine from the extrasynaptic space back into the cell’s releasable pool of dopamine [23]. Immunocytochemistry confirmed the expression of DAT on the embryonic dopaminergic neurons used to generate the TE-NSPs, suggesting they contain the machinery for dopamine re-uptake (Figure 3a). Furthermore, Liu et al. reported that the presynaptic scaffolding proteins bassoon, RIM, and Elk form active-zone-like release sites for dopamine [24]. In particular, within these sites, they found that RIM was essential to dopamine release [24]. Immunocytochemistry labeling of the TE-NSPs confirmed both the expression and co-localization of the scaffolding proteins bassoon and RIM with the dopaminergic neuron marker tyrosine hydroxylase along dopaminergic neurites (Figure 3b). Furthermore, such sites of co-localization appear at points of local thickening of axonal projections, referred to as dopaminergic varicosities. 

In addition to immunocytochemical analysis, functional studies utilizing FSCV were used to examine the capacity of enriched unidirectional TE-NSPs to release dopamine in vitro. At 24–35 DIV, a carbon-fiber electrode was used to record the evoked dopamine release in both the dopaminergic aggregate and at the distal end of the micro-column containing the terminals of the axonal tracts. It was found that dopamine release could be elicited in only the somatic region of unidirectional TE-NSPs (Figure 3c), and evoked dopamine release in the axon terminals was not measurable at these time points. Cyclic voltammograms recorded during dopamine release in the neuronal aggregate region all demonstrated characteristic oxidation between 0.55 and 0.65 V and a reduction between −0.20 and −0.30 V [25]. Under the parameters used in these studies, the pattern of oxidation and reduction may be used to accurately identify currents produced by the action-potential-dependent release of catecholamines, such as dopamine. Current traces recorded in the aggregate region across several TE-NSPs demonstrated an average peak extracellular dopamine concentration of 279.9 ± 252.8 nM (mean ± standard deviation) immediately following electrical stimulation in the enriched TE-NSPs. 

To further demonstrate the expression of the DAT on the dopaminergic neurons, the evoked dopamine release was recorded from enriched TE-NSPs following perfusion with increasing concentrations of the DAT inhibitor GBR12909. Evoked release was recorded at baseline, as well as following perfusion, with the following concentrations of GBR12909: 0.0 µM, 0.02 µM, 0.2, µM 2.0 µM, and 20 µM. The application of the DAT inhibitor resulted in higher maximum concentrations of evoked dopamine release and longer release durations (Figure 3d,e). During the initial application, the maximum concentration of evoked dopamine rose rapidly over 0.02–2.0 µM GBR12909; however, the maximum concentration of dopamine only increased modestly from 2.0–20 µM GBR12909, suggesting a plateau in the effect. We also labeled the enriched TE-NSPs with markers of excitatory and inhibitory neurons to ascertain the phenotype of the non-dopaminergic neurons. We found that, in addition to the dopaminergic neurons, the enriched population contained both glutamatergic and GABAergic neurons (Figure 3f).

### 2.3. Functional Synapse Formation with Striatal Population in TE-NSP Test Bed

**Figure 4:**
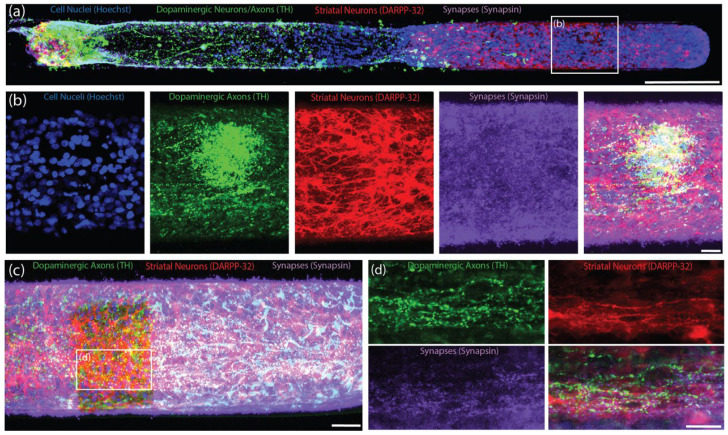
Bidirectional TE-NSPs with Surrogate Striatal Neuron End Targets. Representative confocal reconstructions of TE-NSPs at 14 DIV labeled via immunocytochemistry to denote dopaminergic neurons/axons (TH, green), striatal medium spiny neurons (DARPP-32, red), and synapses (synapsin, purple), with nuclear counterstain (Hoechst, blue). (**a**) An enriched TE-NSP (dopaminergic neurons on the left side) plated with an aggregated striatal end target (located on the right side). (**b**) Higher-magnification reconstructions from a demonstrative region in (**a**) depicting dense dopaminergic neurite outgrowth and containing a high degree of synapsin labeling (purple) within the striatal aggregate. (**c**) A striatal aggregate end target within a bidirectional, enriched TE-NSP. (**d**) Higher-magnification reconstructions from a demonstrative region in (**c**) depict synapsin+ puncta (purple) decorating putative dendrites projecting from striatal neurons (red) shown with dopaminergic axonal varicosities (green). Scale bar (**a**) = 250 µm. Scale bar (**b**) = 20 µm. Scale bar (**c**) = 50 µm. Scale bar (**d**) = 25 µm.

As the dopaminergic axons comprising the nigrostriatal pathway synapse with striatal neurons in the brain, we probed the ability of our TE-NSP axons to synapse with a population of striatal neurons in vitro. TE-NSPs were generated and, after 3–10 DIV, embryonic rat striatal aggregates were inserted into the vacant ends of the micro-columns. After 14 DIV, immunocytochemistry was performed in order to assess potential synaptic integration between the two populations. This analysis confirmed the presence of the appropriate neuronal sub-types in the two aggregate populations, specifically TH+ dopaminergic neurons and DARPP-32+ medium spiny striatal neurons (Figure 4a–c). Moreover, confocal microscopy revealed extensive axonal–dendritic integration and putative synapse formation involving the dopaminergic axons and striatal neurons (Figure 4b–d). In some instances, neurites from enriched dopaminergic aggregates demonstrated a concentrated network of outgrowth within the striatal aggregate, representative of the dense arborization of dopaminergic axons in the striatum in vivo, albeit in a highly localized fashion (Figure 4b). 

**Figure 5:**
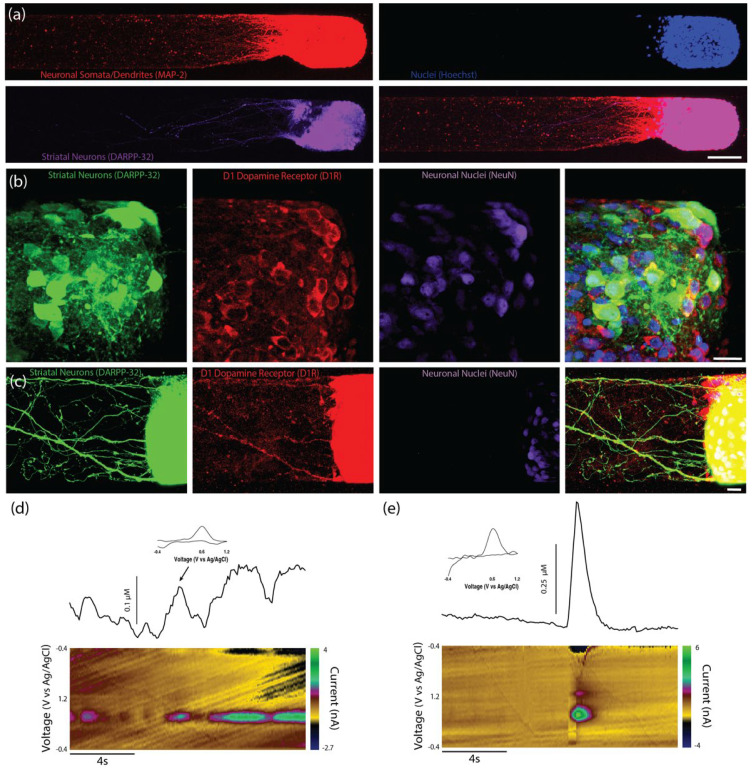
Composition of surrogate striatal neuron end targets and functionality of bidirectional TE-NSPs. (**a**) Representative confocal reconstruction at 14 DIV showing a striatal neuronal end target labeled via immunocytochemistry to denote neuronal somata and dendrites (MAP-2, red) and medium spiny neurons (DARPP-32, purple), with nuclear counterstain (Hoechst, blue). (**b**,**c**) Representative confocal reconstruction at 14 DIV of a striatal aggregate end target within a bidirectional, enriched TE-NSP. The striatal aggregate was labeled via immunocytochemistry to denote striatal neurons (DARPP-32, green), the D1 dopamine receptor (D1R, red), and neuronal nuclei (NeuN, purple) with nuclear counterstain (Hoechst, blue). As expected, striatal neurons express the dopamine D1 receptor on their cell bodies, as well as along their neurites. (**d**,**e**) FSCV was used to measure dopamine release from the striatal end of bi-directional TENSPs (n = 6). Representative color plots displaying the current recorded as the potential of the carbon fiber electrode were linearly scanned from −0.4 to 1.2 V and back to −0.4 V vs. Ag/AgCl every 100 ms (bottom). Cross-sections from the color plots display individual cyclic voltammograms exhibiting an oxidation peak at 0.65 V and a reduction trough at −0.3 V, which are characteristic of dopamine (inset). Representative time vs. concentration plots of the evoked dopamine release (top). (**d**) Intrinsic dopamine release was recorded from striatal aggregates within bidirectional enriched dopaminergic TE-NSPs; the carbon-fiber recording electrode was placed within the striatal aggregate. (**e**) Evoked dopamine release was also recorded from striatal aggregates within bidirectional enriched dopaminergic TE-NSPs; the stimulating electrode was positioned to span the striatal aggregate while the carbon-fiber recording electrode was placed within the striatal aggregate. Scale bar (**a**) = 100 µm. Scale bar (**b**,**c**) = 20 µm.

Immunocytochemistry confirmed that the majority of the striatal (DARPP-32+) neurites were also MAP-2+, suggesting that they were dendrites (Figure 5a). Immunocytochemistry also confirmed that both the striatal neuron cell bodies and neurites expressed the dopamine receptor D1R (Figure 5b,c). 

While we were unable to record the dopamine release in the axonal region of unidirectional TE-NSPs, we tested the hypothesis that the presence of the surrogate striatal end target would enhance dopamine release from the dopaminergic axon terminals. At 35 DIV, enriched bidirectional TE-NSPs containing surrogate striatal end targets underwent FSCV; initially, the carbon fiber recording electrode was placed within the striatal aggregate, and recordings were taken without electrical stimulation. In certain instances, intrinsically released dopamine (mean 113 nM) was recorded within the surrogate striatal end target (Figure 5d). Cyclic voltammograms recorded during dopamine release demonstrated characteristic oxidation between 0.55 and 0.65 V and a reduction between −0.20 and −0.30 V [25]. Next, the bipolar stimulating electrode was used to locally assess the evoked dopamine release. It was found that dopamine release could be electrically evoked within the striatal aggregate, indicating measurable dopamine release from the axonal terminals projecting from the dopaminergic neuronal end of the TE-NSP. Here, current traces were recorded within the striatal aggregate across multiple TE-NSPs and demonstrated a mean peak extracellular dopamine concentration of 374.6 ± 108.0 nM (mean ± standard deviation) immediately following electrical stimulation (Figure 5e).

### 2.4. Establishing Optically-Cleared Thick-Tissue Histological Techniques

**Figure 6:**
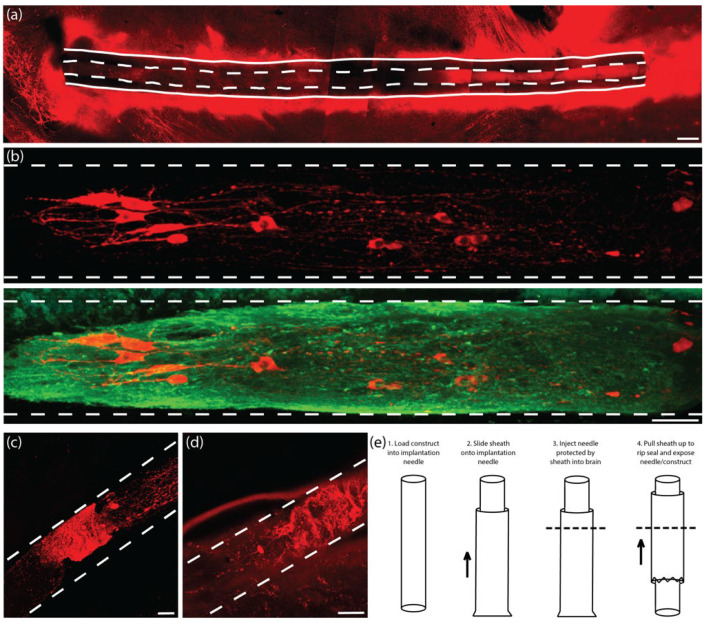
Development of histological and transplantation methodologies. In comparison to conventional sectioning methods, the use of tissue clearing allowed for complete visualization of the TE-NSPs in vivo while avoiding damage to the TE-NSPs during traditional thin sectioning. (**a**) Representative confocal reconstruction of an enriched TE-NSP immediately following implantation labeled via immunocytochemistry to denote dopaminergic neurons/axons (TH, red). Tissue clearing enabled a global perspective on implant integrity and trajectory. (**b**) Representative confocal reconstruction of an enriched TE-NSP immediately following implantation labeled via immunocytochemistry to denote dopaminergic neurons/axons (TH, red) and all neurons/axons (β-tubulin III, green). Structures as small as neuronal somata and individual neurites could be imaged with ease using tissue clearing in conjunction with confocal microscopy. (**c**,**d**) Representative confocal reconstructions of enriched TE-NSP immediately following implantation labeled via immunocytochemistry to denote dopaminergic neurons/axons (TH, red). The original delivery process was found to occasionally damage the TE-NSP neurons and axonal projections due to the delivery needle coring out brain tissue. In these two examples, the TE-NSP axons within the lumen were compressed due to forces from cored brain tissue pushed into the needle (each large TH+ mass shows undulated axons within the lumen). (**e**) In order to prevent the compression of the TE-NSP by the brain during implantation, a needle sheath with a breakable seal was used to cover the end of the needle. This methodology consisted of the following steps: (1) The construct was loaded into the implantation needle; (2) once loaded, the sheath was slid onto the needle and completely covered the end of the needle (not shown); (3) the whole apparatus (needle and sheath) was lowered into the brain to the correct depth (the dashed line represents the brain surface); and (4) forceps were used to pull the sheath upwards, which breaks the seal on the end of the needle and left the construct exposed at the correct depth in the brain. As with the original methodology, a plunger was used to hold the TE-NSP in place while the needle and sheath were removed from the brain, thereby depositing the intact TE-NSP in the wake of the needle. Scale bar (**a**) = 200 µm. Scale bar (**b**) = 50 µm. Scale bar (**c**,**d**) = 50 µm.

Conventional histological techniques, including sectioning paraffin-embedded tissue and cryosectioning frozen tissue, sometimes damaged implanted TE-NSPs and/or pulled implanted TE-NSPs from within the agarose micro-columns. Furthermore, these sections did not have sufficient depth to enable continuous visualization of an implanted TE-NSP from the SNpc to the striatum. Therefore, conventional techniques prevented global characterization of the TE-NSPs (e.g., the accuracy of the implantation trajectory and maintenance of axonal cytoarchitecture spanning the micro-column). To avoid these drawbacks, we applied histological techniques involving tissue clearing that allowed us to label large sections of tissue. We took 2 mm wide sagittal sections from the implanted animals that contained the TE-NSPs fully encapsulated within sections. Next, we combined two previously published clearing methods (iDISCO and Visikol) to optimize antibody penetration and minimize the use of toxic chemicals. It was found that this clearing protocol allowed for the characterization of the TE-NSPs on both macro and micro scales (Figure 6a,b). This technique enabled us to discern the implantation trajectory of the construct and determine whether the internalized axonal cytoarchitecture spanned the length of the micro-column. Importantly, it also allowed us to visualize small structures such as neuronal somata and individual neurites (Figure 6b). 

### 2.5. Establishing Implantation Methodology

Following the establishment of our histological techniques, we performed multiple TE-NSP implantations followed by the immediate sacrifice of the animals to ascertain the efficacy of our implantation methodology. In several instances, evidence of aggregate and axonal compression within the micro-column lumen was observed (Figure 6c,d). Further investigation determined that although the implantation needle has a small inner diameter (420 µm), some brain tissue could press into the needle opening, which then compacted the TE-NSP within the needle. 

In order to prevent compression of the constructs, we developed a method to use a “sheath” to cover the end of the implantation needle during insertion but allow for TE-NSP ejection at the correct depth within the brain (Figure 6e). The sheath was fabricated by sealing the end of a tube (inner diameter: 559 µm; outer diameter: 686 µm) using an electrical current. During implantation, the sheath was slid onto the end of the implantation needle after the TE-NSP was loaded into the needle. The sheath stayed in place, protecting the end of the needle and preventing coring of the brain, while the needle was stereotactically lowered to the correct location within the brain. Finally, forceps were used to pull the sheath upwards, which broke the seal on the end of the needle and exposed the needle/TE-NSP within the brain. A guide wire placed within the needle was used as a plunger to hold the TE-NSP in place as the needle and sheath were withdrawn from the brain. The sheath added approximately 150 µm to the diameter of the implantation needle, keeping the overall footprint under 700 µm in diameter.

### 2.6. Validation of PD Model of 6-OHDA Lesion of the SNpc in Athymic Rats

**Figure 7:**
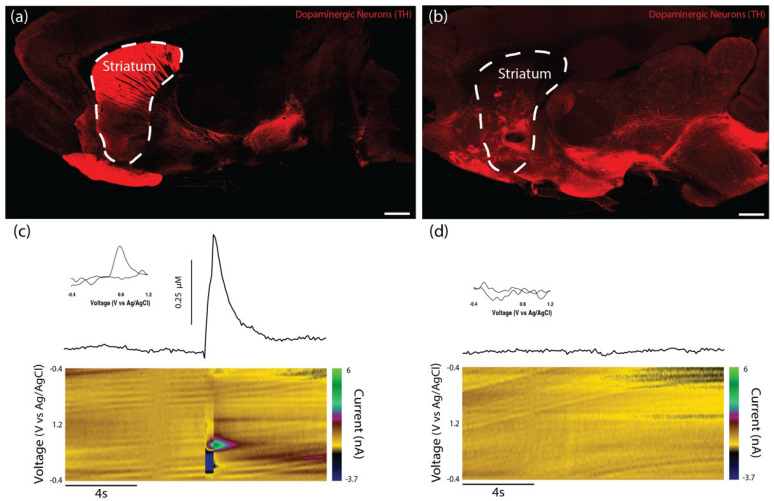
Validation of PD model in athymic rats featuring a 6-OHDA lesion of the SNpc. Athymic rats lesioned by the dopaminergic neurotoxin 6-OHDA injected into the SNpc were used as surgical subjects for the in vivo studies. (**a**) Representative confocal reconstruction of the non-lesioned side of the rat brain labeled via immunocytochemistry to denote dopaminergic neurons/axons (TH, red). As expected, the non-lesioned side demonstrated significant TH labeling within the striatum. (**b**) Representative confocal reconstruction of the lesioned side of the rat brain labeled via immunohistochemistry to denote dopaminergic neurons/axons (TH, red). The lesioned side of the brain demonstrated minimal TH labeling in the striatal region—especially in dorsal striatum—validating the efficacy of the 6-OHDA lesion in destroying dopaminergic neurons in the SNpc and their axons within the nigrostriatal pathway. (**c**,**d**) Evoked dopamine release was investigated in slices from both the non-lesioned and lesioned sides of the brain using FSCV. (**c**) Representative color plot (bottom), cyclic voltammogram (inset), and time vs. concentration plot (top) of the evoked dopamine release recorded in the non-lesioned side of the brain (n = 3) as potential of the carbon fiber electrode was linearly scanned from −0.4 to 1.2 V and back to −0.4 V vs. Ag/AgCl every 100 ms. The cyclic voltammogram exhibited an oxidation peak between 0.55 and 0.65 V and a reduction trough between −0.2 and −0.3 V, which are characteristic of dopamine. The maximum concentrations of dopamine released by the non-lesioned side were over 300 nM. (**d**) Representative color plot (bottom), cyclic voltammogram (inset), and time vs. concentration plot (top) of the evoked dopamine release recorded in the lesioned side of the brain (n = 3). The cyclic voltammogram did not exhibit peaks or troughs representative of dopamine, and no discernable concentration of dopamine was recorded. These findings validated the functional consequences of the 6-OHDA lesion in destroying dopaminergic neurons/axons of the nigrostriatal pathway. Scale bar (**a**,**b**) = 1 mm.

Charles River Laboratories (Raleigh, North Carolina) offer a model of PD featuring a unilateral 6-OHDA lesion of the SNpc in athymic (T-cell deficient) rats. However, while the presence of motor deficits was confirmed by the vendor prior to shipment, the degeneration of striatal dopaminergic projections and loss of striatal dopamine tone in this model had not previously been validated. Therefore, we used histological and functional analyses to determine whether this model demonstrated a lack of dopaminergic innervation of the striatum. Immunohistochemistry revealed dense TH labeling in the striatum of the unlesioned side of the brain, while the lesioned side displayed minimal TH labeling, especially in the dorsal striatum (Figure 7a,b). While the 6-OHDA lesion appeared to ablate the vast majority of the dopaminergic staining within the SNpc of the lesioned side, a few sparse patches of staining could be found. We also performed FSCV in the striatum to confirm that dopamine release could not be evoked by electrical stimulation in the lesioned side of the brain. No discernable dopamine signal was found within the dorsal striatum of the lesioned side, whereas the dorsal striatum on the unlesioned side demonstrated significant evoked dopamine release of >300 nM in all cases (Figure 7c,d). 

### 2.7. Structural Analyses following TE-NSP Implantation into 6-OHDA Lesioned Athymic Rats

**Figure 8:**
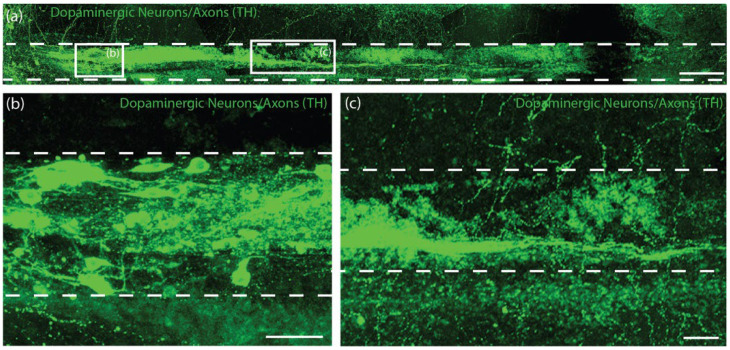
TE-NSP neuronal survival at 1-week post-implant. (**a**) Representative confocal reconstruction of an enriched TE-NSP 1-week post-implant labeled via immunohistochemistry to denote dopaminergic neurons/axons (TH, green). The TE-NSPs maintained their cytoarchitecture consisting of distinct cell body and neurite regions, with dopaminergic neurites projecting longitudinally within the micro-column. Higher magnification reconstructions from demonstrative regions in (**a**) depict the (**b**) dopaminergic neuron aggregate and (**c**) robust, aligned TH+ axons. At 1-week in vivo, neuronal survival was found within all of the transplanted TE-NSPs; however, no evidence of neuronal survival was found within the other transplant groups (solitary aggregates transplanted into the SNpc or striatum). Scale bar (**a**) = 250 µm. Scale bar (**b**,**c**) = 50 µm.

We sought to characterize survival, maintenance of cytoarchitecture, and dopaminergic outgrowth following TE-NSP implantation into the rat brain. Using the previously optimized transplantation methodology, purified GFP+ TE-NSPs that exhibited >5 mm in axonal outgrowth by 21 DIV were stereotactically micro-injected to approximate the nigrostriatal pathway in the athymic, unilateral 6-OHDA rat model. Other transplantation groups, including solitary aggregates, aggregates within micro-columns without pre-grown axonal projections (implanted at 1–3 days post-plating), acellular micro-columns containing collagen, and impure TE-NSPs (non-FACS purified) served as control groups. Animals were sacrificed at either 1-week or 1-month time points, after which their tissue was sectioned, labeled, and cleared using our previously optimized histological techniques. Our findings confirmed that the optimized implantation method featuring an outer protective sheath prevented compression of the TE-NSPs and was capable of precisely delivering TE-NSPs to span the nigrostriatal pathway in this rat model. Indeed, enriched TE-NSPs demonstrated distinct neuronal somata and axonal zones that showed no signs of compaction (Figure 8a). At the 1-week time point, implanted neuronal survival and maintenance of pre-formed axonal tracts were observed in all of the animals receiving TE-NSPs (100%), appearing as a distinct cluster of GFP+ neurons with defined morphologies within the lumen of the micro-column. However, evidence of surviving implanted cells was significantly reduced when neuronal aggregates alone (absent in the protective micro-column) were implanted into the SNpc or the striatum (0%; *p* < 0.001). Surviving TE-NSP cells were also labeled for the dopaminergic marker TH, confirming the preservation of dopaminergic neuronal populations and their axonal extensions (Figure 8a–c). In particular, longitudinally projecting TH+ axons were present within the lumen of all constructs, demonstrating that the TE-NSPs were able to maintain their cytoarchitecture following transplantation into the brain. However, no evidence of dopaminergic neurite outgrowth from the micro-column was found at this early time point. 

**Figure 9:**
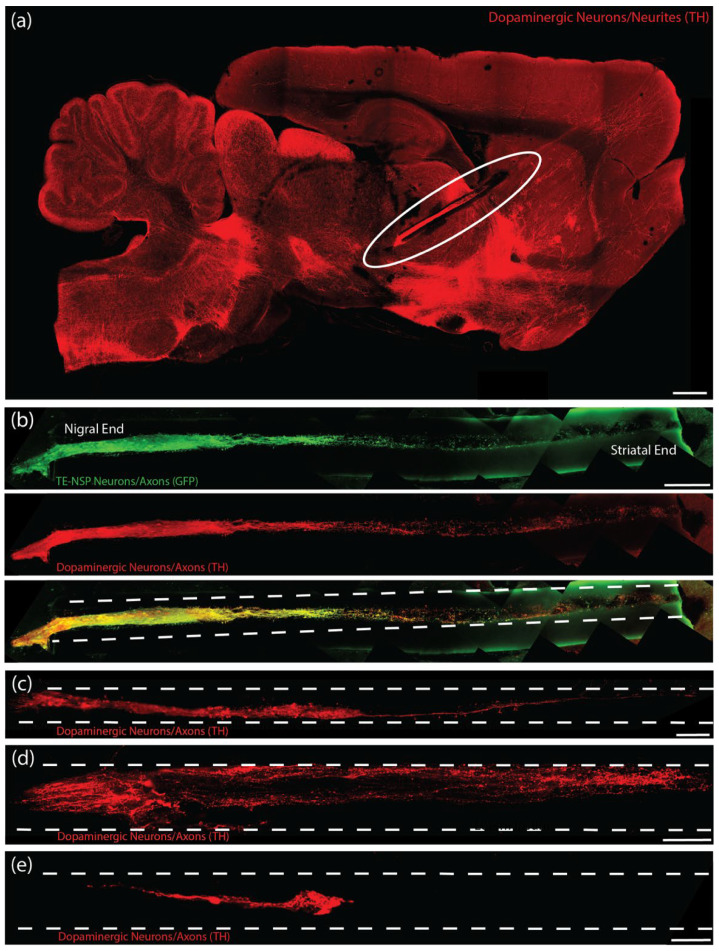
TE-NSP neuronal survival and axon architecture at 1-month post-implant in rat 6-OHDA lesion model of PD. (**a**) Representative confocal reconstruction of an enriched TE-NSP 1-month post-implant labeled via immunocytochemistry to denote dopaminergic neurons/axons (TH, red). The aggregate end of the TE-NSP was positioned in the SN while the axonal end of the TE-NSP terminated in the striatum. Implanted neurons and axons tracts were preserved in lesioned animals. Background has been increased to display the intact physical structure of the brain surrounding the implant and along its implantation path. (**b**) Representative confocal reconstruction of an enriched TE-NSP 1-month post-implant labeled via immunohistochemistry to denote GFP (GFP, green) and dopaminergic neurons/axons (TH, red). (**c**–**e**) Representative confocal reconstructions showing various types of implanted constructs at 1-month post-implant labeled via immunohistochemistry to denote dopaminergic neurons/axons (TH, red). (**c**) Enriched TE-NSPs maintained their cytoarchitecture with discrete somata and axonal regions; they also demonstrated neurites spanning the full length of the micro-column. (**d**) Non-enriched TE-NSPs generally did not exhibit neurites spanning the length of the micro-column. (**e**) Enriched aggregates added to micro-columns 1 day prior to implant did not display long-distance neurite outgrowth within the micro-column. Scale bar (**a**) = 1 mm. Scale bar (**b**) = 250 µm. Scale bar (**c**) = 200 µm. Scale bar (**d**,**e**) = 100 µm.

As we did not observe evidence of surviving neurons from implanted aggregates at the 1-week time point, we adjusted our controls and used the following transplantation groups for the 1-month histological time point: (i) Fully grown, enriched TE-NSPs (implanted at 21 DIV), (ii) aggregates within micro-columns (implanted at 1–3 DIV), (iii) acellular micro-columns containing collagen only, (iv) fully grown, impure TE-NSPs (implanted at 21 DIV), and (v) lesioned, non-repaired animals. We found that TE-NSPs’ integrity and precise trajectory spanning the nigrostriatal pathway were maintained for 1 month (Figure 9a). We also found that the enriched TE-NSPs contained clear evidence of viable neurons in the majority of cases, consistently maintained their axonal cytoarchitecture, and were the only group to exhibit dopaminergic projections spanning the entire length of the micro-column (Figure 9a–c). Indeed, we found that the percentage of enriched TE-NSPs exhibiting neuronal survival (75%) was significantly increased relative to the percentage of the aggregate in micro-column controls exhibiting neuronal survival (33%; *p* = 0.004). Furthermore, the percentage of enriched TE-NSPs containing axons spanning the length of the micro-column (50%) was significantly higher than the percentage of aggregates in micro-column controls with spanning axons (0%; *p* < 0.001) at this time point (Figure 9e). Impure TE-NSPs also had a high rate of cell survival (66%) that did not statistically differ from that of enriched TE-NSPs (*p* = 0.26) and also displayed discrete somatic and axonal regions (Figure 9d). While acellular micro-columns showed the capability to support host neurite ingrowth from the SNpc, such ingrowth measured <250 µm in length. 

**Figure 10:**
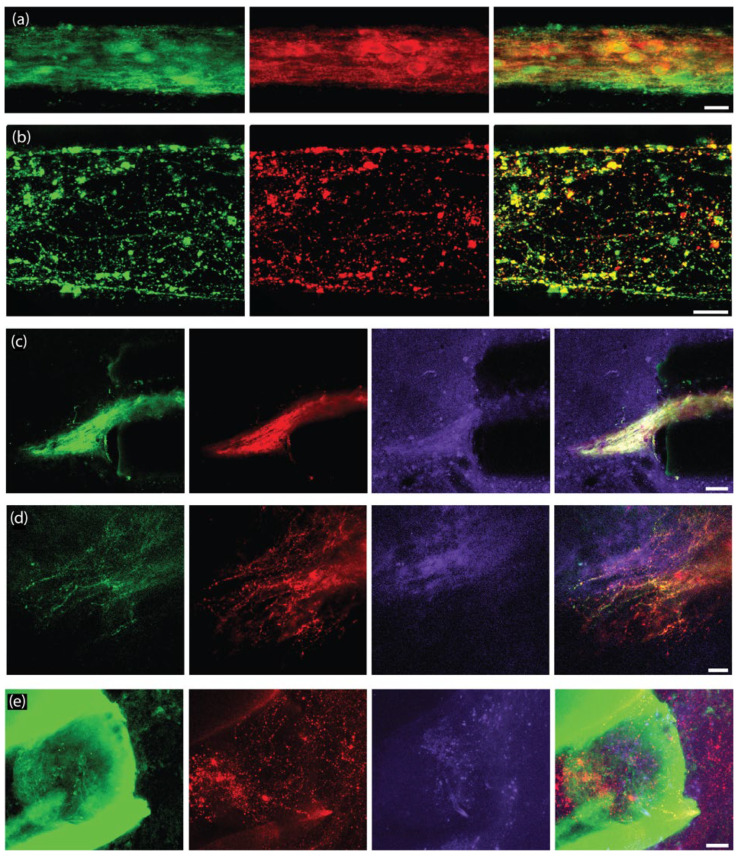
Survival and outgrowth of enriched TE-NSPs at 1-month post-implant in rat 6-OHDA lesion model of PD. Representative confocal reconstructions of implanted enriched TE-NSPs at 1-month post-implant labeled via immunohistochemistry to denote GFP (GFP, green), dopaminergic neurons/axons (TH, red), and synapses (synapsin, purple). (**a**) Dopaminergic neurons within the aggregate region displayed healthy morphologies. (**b**) Dopaminergic neurites maintained healthy projections longitudinally within the tube. (**c**,**d**) TE-NSP neurons exhibited neurite outgrowth into the host SNpc from the aggregate region. In these instances, regions containing outgrowth displayed concentrated synapsin labeling. (**e**) TE-NSPs also exhibited neurite outgrowth into the host striatum from the axonal region. In these instances, thin neurites were observed, projecting from areas of dense TH labeling within the end of the tube; note, in this example, the micro-column is autofluorescing green. Scale bar (**a**,**b**) = 25 µm. Scale bar (**c**) = 50 µm. Scale bar (**d**,**e**) = 25 µm.

At this 1-month post-implant time point, enriched TE-NSPs consistently showed clusters of morphologically healthy neuronal somata at the ventral end of the micro-column within the SNpc (Figure 10a). Moreover, enriched TE-NSPs demonstrated dopaminergic axonal tracts running longitudinally within the lumen of the micro-column (projecting from the SNpc end towards the striatal end); these dopaminergic axons showed typical varicosities—appearing as a “string of pearls”—consistent with clustered dopamine machinery at discrete locations along the shaft of an axon (Figure 10b). Notably, enriched and impure TE-NSPs were the only groups that exhibited dopaminergic neurite outgrowth from the TE-NSP into the SNpc (Figure 10c,d). In addition, enriched TE-NSPs were the only group that demonstrated outgrowth from the TE-NSP axon tracts into the host striatum (Figure 10e). In these instances, numerous TH+ neurites were found projecting out of the micro-column lumen and localized with synapsin labeling, suggesting synaptic integration between TE-NSP axons and host striatal neurons.

### 2.8. Functional Analyses Following TE-NSP Implantation into 6-OHDA Lesioned Athymic Rats

**Figure 11:**
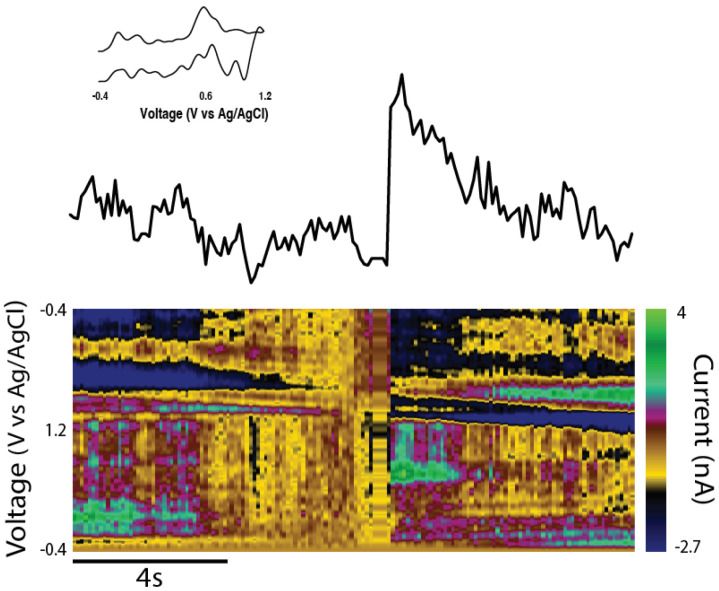
TE-NSPs elicited measurable striatal dopamine in rat 6-OHDA lesion model of PD. Evoked dopamine release was investigated in slices from implanted animals at 1-month in vivo. Color plot (bottom), cyclic voltammogram (inset), and time vs. concentration plot (top) of evoked dopamine release recorded in the striatum of an animal implanted with an enriched TE-NSP. The color plot exhibited an oxidation peak between 0.55 and 0.65 V and a reduction trough between −0.2 and −0.3 V, which are characteristic of dopamine. The maximum concentration of dopamine released by the implanted slice was approximately 95 nM following electrical stimulation. The data shown are an average of three collections, and the stimulation artifact was removed.

In order to assess the efficacy of TE-NSP implants in lesioned athymic rats, FSCV was performed on a subset of the transplant groups at the 1-month time point. Transplanted animals were sacrificed, their brains were rapidly harvested, blocked in the sagittal plane, and a vibratome was used to cut 400 µm thick brain sections that longitudinally contained the TE-NSP. A stimulating electrode was used to evoke dopamine release, and a carbon-fiber electrode was used to record the release within the dorsal striatum of the slice. At the 1-month time point, evoked dopamine release was compared between brain sections from lesioned animals either receiving enriched TE-NSPs, acellular tubes, or no implant. Here, FSCV revealed that the enriched TE-NSPs were the only group to exhibit any discernable dopamine signal in the striatum, as shown by a standard color plot displaying stereotypical dopamine oxidation and reduction troughs (Figure 11). Here, the dopamine signal was recorded in the dorsal striatum and demonstrated a release concentration of approximately 95 nM, within the minimum range of 50–100 nM suggested to correspond to improvements in motor function [9]. For comparison, striatal dopamine levels following TE-NSP implantation clearly exceeded those of non-repaired animals (no measurable dopamine in any animals; refer to Figure 7d), but at this relatively early time point did not approach the levels found in the striatum of a non-lesioned hemisphere (>300 nM in all cases; refer to Figure 7c). 

## 3. Discussion

Current PD treatments, including the use of dopamine-replacement strategies and DBS, are aimed at reducing motor disabilities rather than correcting the underlying cause of motor system dysfunction. Furthermore, while dopaminergic neuron and/or fetal graft implants into the striatum may provide a local source of dopamine, these approaches do not recreate the nigrostriatal circuit and thus may not provide appropriate regulation of dopamine release. To address these gaps, we are developing a tissue-engineered solution that may be precisely delivered to physically restore lost dopaminergic neurons in the SNpc and their axonal projections to the striatum. To achieve this objective, we built upon our previously developed microtissue engineering platform to generate TE-NSPs with a neuronal population comprised of over 60% dopaminergic neurons. These TE-NSPs exhibited the target cytoarchitecture of discrete somatic and axonal regions, with axons projecting the length of the tube. The use of dopaminergic cell aggregates, in combination with an extracellular matrix (collagen) core, supported dopaminergic axonal projections measuring over 5 mm in length by 21 DIV and displaying a mean cell viability of >85% at 14 DIV. Thus, these studies demonstrated the feasibility of generating healthy, purified TE-NSPs with sufficient length to span the nigrostriatal pathway in a rat model of PD. 

Unidirectional TE-NSPs expressed machinery necessary for dopamine release and re-uptake. Specifically, immunocytochemical labeling demonstrated the presence of the dopamine transporter responsible for dopamine recycling, as well as two scaffolding proteins necessary for dopamine release from active zone-like sites on dopaminergic varicosities [24]. Furthermore, FSCV confirmed the ability to evoke dopamine release within the dopaminergic aggregate region of enriched TE-NSPs and cyclic voltammograms demonstrated oxidation and reduction potentials characteristic of dopamine [25]. The application of the dopamine transporter (DAT) inhibitor GBR12909 also confirmed the presence of functional DAT for dopamine recycling within the TE-NSPs. These studies demonstrated an increase in the amplitude and half-life of evoked dopamine following perfusion with GBR12909, consistent with other studies blocking dopamine re-uptake [26,27]. Interestingly, the peak concentration of evoked dopamine initially rose rapidly upon increasing the concentration of GBR12909 from 0.0 µM to 2 µM, after which it plateaued as the concentration of GBR12909 approached 20 µM. This diminishing release likely occurred because 20 µM of GBR12909 either blocked all the re-uptake locations or was harmful to the dopaminergic neurons. 

In order to further characterize the function of the TE-NSPs, we created an in vitro test bed consisting of TE-NSPs containing a surrogate end target comprised of striatal neurons. The test bed revealed extensive presynaptic puncta labeling in areas where the TE-NSP axons grew into the striatal neurons, and the striatal neurons also expressed dopamine receptors along their cell bodies and neurites. In fact, in some instances, the striatal aggregate appeared to recruit dense dopaminergic axonal outgrowth and arborization. These findings suggest that the dopaminergic axons received cues from and integrated with the striatal neuron population, providing evidence of the functionality of TE-NSPs in vitro. Furthermore, while we were unable to record the evoked dopamine release in the axon terminals of unidirectional TE-NSPs at the time points interrogated, we were able to record dopamine from TE-NSP axons within the striatal end target of the test bed. Current traces recorded in the striatal aggregate across several TE-NSPs demonstrated an average peak extracellular dopamine concentration of ~375 nM immediately following electrical stimulation, which is similar to the estimated tonic concentration of dopamine in the striatum of rats [28]. It is possible that the presence of a synaptic target and synapse formation was necessary for dopaminergic axons to upregulate the necessary machinery for dopamine release. Consistent with this explanation, autaptic synapses would be responsible for the evoked release from the dopaminergic aggregates. However, it is also possible that axon terminals within unidirectional TE-NSPs were simply not concentrated enough to create a detectable dopamine signal. The striatal end target could have provided an impetus for dense axonal outgrowth capable of generating a larger dopamine signal. Regardless, our ability to record the dopamine release in the striatal end target, and not in the axon terminals of unidirectional TE-NSPs, provides evidence that functional integration occurred with the striatal neurons.

In addition to fabricating and characterizing TE-NSPs in vitro, a goal of the current study was to establish a proof of concept that implanted TE-NSPs could survive and integrate in vivo. However, several additional techniques needed to be established prior to in vivo testing. For instance, the framework to assess the performance of the constructs needed to be established. Conventional histological techniques did not enable sufficient micro- to macro-scale visualization to fully appreciate TE-NSPs’ implant trajectory, maintenance of neuronal–axonal architecture, and neurite outgrowth into host tissue. Therefore, multiple established tissue-clearing and -labeling protocols were combined to create an optimized histological method capable of revealing both global and minute TE-NSP structures. Furthermore, the capability to stereotactically transplant the constructs to span the correct brain regions without damaging the TE-NSPs required demonstration. The development of the needle “sheath” was instrumental in ensuring that the insertion process did not compress the TE-NSP cytoarchitecture within the tube. Lastly, the athymic rat model featuring a unilateral 6-OHDA lesion of the SNpc required validation to confirm that it exhibited sufficient loss of the nigrostriatal pathway and loss of dopaminergic innervation within the striatum to underpin the PD-like motor dysfunction. The performance of the chosen histological and functional metrics both verified that the model showed PD-like degeneration and functional deficits, while also supporting the use of these outcome metrics in subsequent testing of TE-NSP efficacy using this model. 

The performance of the TE-NSPs in the in vivo rat PD model was compared to contemporary regenerative medicine strategies for the treatment of PD, including dopaminergic neurons implanted into the SNpc, dopaminergic neurons implanted into the striatum, and acellular biomaterial scaffolds. At the 1-week time point, immunohistochemistry revealed robust implanted neuronal survival and the maintenance of axonal cytoarchitecture in all animals receiving enriched TE-NSPs. In contrast, solitary aggregates implanted into the SNpc and striatum did not exhibit surviving neurons at the 1-week time point, which could be attributed to the lack of a protective biomaterial encasement during the insertion process and the low cell densities in these implants. It is important to note that the neuronal density of the TE-NSPs, striatal-only implants, and nigral-only implants was the same; however, alternative implant strategies in the striatum or nigra typically utilize close to 100 times more cells to address the severe neuronal attrition common in these implant paradigms [29]. The combination of TE-NSPs being mature, pre-formed neural networks and the biocompatible encasement may provide a degree of protection from potentially detrimental aspects of the host micro-environment during and immediately following transplantation. This hypothesis is supported by the higher survival rates of the aggregates implanted within micro-columns; however, a limitation of this study was the relatively low number of aggregate-only implants performed in this study (n = 4 in total). The physical support and bioactive signaling provided by the extracellular matrix within the micro-columns could have also improved cell survival and integration in vivo. Regardless, the improved survival of TE-NSP neurons in comparison to neuronal aggregates alone demonstrates the superiority of tissue-engineered therapies in general and TE-NSPs in particular in comparison to purely cell-based treatments for PD. 

At the 1-month time point, immunohistochemistry revealed that the enriched TE-NSPs surpassed other transplantation groups containing dopaminergic neurons, exhibiting higher rates of survival (75%), neuronal density, axonal density, and neurite outgrowth. In particular, enriched TE-NSPs demonstrated a 3- to 5-fold increase in mean neuronal and axonal density, respectively, in comparison to aggregates implanted within micro-columns; however, future transplant studies will require an increase in the number of animals per treatment group. Enriched TE-NSPs were also the only group to exhibit projections spanning the length of the micro-column and dopaminergic neurite (presumably axonal) outgrowth into the striatum. Fully grown impure TE-NSPs also qualitatively outperformed other groups but showed a decreased density of dopaminergic neurons/axons in comparison to the enriched TE-NSPs. As we specifically labeled dopaminergic neurons/axons, it is likely that the initial low purity of the impure TE-NSPs was responsible for this trend. In previous work, we found that TH+ outgrowth only represented approximately 65% of the total axonal length in impure TE-NSPs [19]. This phenomenon could be responsible for the lack of dopaminergic projections spanning the micro-column within the impure group. Aggregates implanted within micro-columns (delivered at 1–3 DIV) demonstrated lower rates of survival (although superior to aggregate-only implants observed at 1-week post-implant) and little dopaminergic outgrowth within the micro-columns. It is possible that the in vivo environment did not provide the necessary trophic support to these embryonic neurons in their growth phase and/or that any growth signals were not directed appropriately. In comparison, TE-NSPs in culture were provided with basic fibroblast growth factor (4 ng/mL), albeit absent of any geometric gradient. Lastly, while acellular micro-columns containing collagen demonstrated the ability to support dopaminergic neurite ingrowth from the host, the short length of this ingrowth (<250 µM) could likely be attributed to the low number of residual dopaminergic neurons present within the lesioned animals in addition to the limited intrinsic capacity of CNS axons to regenerate. These findings showcase the advantages of TE-NSP technology by illustrating that the TE-NSPs uniquely showed the capacity to provide dopaminergic neuronal replacement and targeted axonal reconnection across the SNpc and striatum. Furthermore, as TE-NSPs are pre-fabricated in vitro with axon tracts spanning the distance from the SNpc to the striatum at the time of implant, they should be able to integrate with the host brain soon after transplantation. In contrast, dopaminergic transplantations into the SNpc require significant time in vivo to extend processes to initially reach the striatum while also generally not having directed guidance (potentially leading to off-target innervation). While our findings of TE-NSPs’ neuronal survival, the maintenance of internal axonal tracts, and neurite integration with the host tissue at 1-month post-implant were promising, follow-up studies at chronic time points will be critical to assessing the longevity of the implants and the extent of axonal arborization attained throughout the striatum.

Neurophysiological testing at 1-month post-implant also revealed promising early indications of functional recovery. For instance, enriched TE-NSPs were the only group to demonstrate an evoked dopamine release at the 1-month time point. Here, the cyclic voltammogram displayed typical oxidation and reduction troughs that are characteristic of dopamine, and the concentration of evoked dopamine fell within the desired threshold of 50–100 nM [28,30]. These results show the feasibility of using the TE-NSPs to improve dopamine tone in the striatum and, taken together with the histological evidence, suggest that TE-NSPs could be used to reconstruct the nigrostriatal pathway and augment dopaminergic innervation to the striatum. However, while our results suggest that nascent innervation from TE-NSP axons is the source of the measured dopamine in the dorsal striatum, additional techniques may be necessary to confirm specificity; for instance, using TE-NSPs transduced with opsins and demonstrating light-based stimulation and subsequent dopamine release. In addition, while we would not expect to find complete behavioral recovery at this early 1-month time point—the striatum is a large brain structure and full motor recovery will likely require extensive dopaminergic innervation over many months in vivo—the early signs of dopaminergic signaling are promising. Indeed, the current findings provide a strong rationale for subsequent follow-up studies examining the behavioral consequences—related to motor function in particular —in animals transplanted with TE-NSPs in comparison to control animals, for instance, those receiving acellular micro-columns, striatal-only neuronal implants, and/or no treatment. These studies will likely require longer-term functional testing (i.e., 3–6 months) to assess the levels of functional recovery attained with TE-NSPs in comparison to other implant paradigms.

As noted, TE-NSPs maintained their overall cytoarchitecture in vivo comprised of a discrete population of dopaminergic neurons in the SNpc and bundled, long-projecting axonal tracts leading to the striatum. The long axons present within the TE-NSPs may enable the signaling between the repaired SNpc and striatum to occur through a minimal number of synapses. Such a paradigm closely mimics the structure of the nigrostriatal pathway in vivo and should support appropriately timed dopamine signaling within the striatum in response to afferent signals to the SNpc [13]. In contrast, ectopic transplantations of dopaminergic neurons into the striatum may be unable to “close the loop” to restore endogenous regulation of dopamine signaling due to their incorrect anatomical location [13].

It is possible that further improvements may need to be made to the transplantation paradigm to improve the long-term health of the TE-NSPs. The environment of the brain is generally considered to be hostile upon transplantation, and future exploratory studies should assess the implanted tissue for evidence of blood, hypoxia, ischemia, inflammation, excitotoxicity, reactive astrogliosis, microglia/macrophage activation, and the formation of a glial scar. As we observed complete survival of TE-NSPs at the 1-week time point and the diameter of the aggregates within the TE-NSPs was sufficiently small (<180 µm) to allow for mass transport via diffusion in vivo, we do not believe that hypoxia within the constructs had occurred. In the case of reactive astrocytes, nonsteroidal anti-inflammatory inhibitory drugs (NSAIDs) have been shown to reduce astrogliosis and decrease the upregulation of the glial fibrillary acidic protein (GFAP) in both mice and in vitro models [31]. Alternatively, treatment with inhibitors of histone deacetylases (HDAC), such as valproic acid, has been shown to reduce inflammation mediated by both reactive astrocytes and microglia [31,32,33]. Both valproic acid and NSAIDs are already used clinically, effectively cross the blood–brain barrier (BBB), and would not require local release at the transplantation site [34]. In order to combat the formation of a glial scar, we could also engineer the micro-column tube/hydrogel shell to provide controlled delivery of chondroitinase ABC—an enzyme shown to break down the chondroitin sulfate proteoglycans that represent a large component of the glial scar [35,36]. The controlled release could also be used to provide the dopaminergic neurons with growth factors if we determine that TE-NSPs require enhanced trophic support upon transplantation for sustained viability. Many hydrogels, including agarose, fibrin, collagen, and alginate, have already successfully been used for controlled release within the nervous system of animal models [37,38,39]. Similarly, the degradation rate of the micro-column biomaterial (whether agarose or another hydrogel) could be adjusted if necessary to improve long-term TE-NSP health. As shown in the current study, the 1% agarose micro-columns did not show signs of degradation at the 1-month time point. While the micro-column exterior presumably provides the TE-NSP neurons/axons with protection during and immediately following transplantation, alternative degradation rates may be beneficial for long-term performance by potentially removing a source of ongoing inflammation and enabling more thorough integration with the host at earlier time points.

The current studies establish an important foundation for follow-up longitudinal testing by demonstrating the consistent growth and functionality of TE-NSPs in vitro, showing their survival and burgeoning outgrowth in vivo, and establishing critical techniques including validating the rat lesion model, establishing implant methodology, and demonstrating a high-resolution/thick-tissue optical clearing immunohistochemistry protocol. However, there are a number of important limitations in the current study. While the 6-OHDA lesioned rat model provided a valuable in vivo environment for the exploration of TE-NSP efficacy, this model does not actually mirror the underlying neuropathology of PD, which is marked by the presence of Lewy bodies/neurites and activated microglia that are believed to be responsible for the degeneration of the dopaminergic neurons. Future studies should transplant TE-NSPs into rat synucleinopathy models of neurodegeneration to assess whether the TE-NSP neurons are affected by the pathogenic mechanisms of PD. Furthermore, while the rat embryonic neuronal source used in the current study is useful for proof-of-concept testing, this biomass would not be suitable for clinical testing. Here, it is likely that a human cell source will be necessary to acquire dopaminergic neurons for TE-NSP fabrication. For instance, TE-NSPs may be built using human embryonic stem cells (e.g., a federally approved line) or induced pluripotent stem cells (iPSCs) with design parameters (e.g., the number of neurons or length of axon tracts) suitable to replace the nigrostriatal pathway in humans [13]. Moreover, the fabrication of TE-NSPs using autologous iPSC sources may enable the construction of patient-specific constructs tailored to their particular extent of degeneration. Lastly, the 1-month post-implant time point explored in the current study is too early to expect widespread axonal arborization in the striatum and complete amelioration of motor deficits in this rat model; as such, future studies should explore the long-term efficacy of this strategy. Therefore, future studies will need to validate the growth, structure, and functionality of TE-NSPs built using human dopaminergic neurons, as well as their chronic survival and efficacy upon implant into appropriate preclinical models of PD.

## 4. Materials and Methods

All procedures were approved by the IACUCs at the University of Pennsylvania and the Corporal Michael J. Crescenz Veterans Affairs Medical Center and were carried out in accordance with the Public Health Service Policy on Humane Care and Use of Laboratory Animals (2015).

### 4.1. Three-Dimensional TE-NSP Fabrication

All supplies were from Invitrogen (Waltham, MA, USA), BD Biosciences (Franklin Lakes, NJ, USA), or Sigma-Aldrich (St. Louis, MO, USA) unless otherwise noted. TE-NSPs were comprised of an agarose (Sigma; A9539) ECM hydrogel molded into a cylinder through which axons could grow (see Figure 1). The outer hydrogel structure consisted of 1% agarose in Dulbecco’s phosphate-buffered saline (DPBS). The agarose cylinder, with an outer diameter of 398 µm, was generated by drawing the agarose solution into a capillary tube (Drummond Scientific, Broomall, PA, USA) via capillary action. An acupuncture needle (diameter: 160 µm) (Seirin, Shizuoka, Japan) was inserted into the center of the agarose-filled capillary tube in order to produce an inner column. Cured micro-columns were pushed out of the capillary tubes using a 30-gauge needle (BD; 305128) and placed in DPBS where they were cut to 6–12 mm in length and sterilized under UV light (1 h). Five microliters of the appropriate ECM cocktail were added to each micro-column. ECM cocktails included (1) rat tail type 1 collagen, 1.0 mg/mL; (2) rat tail type I collagen, 1.0 mg/mL, mixed with mouse laminin, 1.0 mg/mL; (3) mouse laminin, 1.75 mg/mL; and (4) rat tail type 1 collagen, 1.0 mg/mL, in 11.70 mM N-(3-Dimethylaminopropyl)-N’-ethylcarbodiimide hydrochloride, 4.3 mM N-Hydroxysuccinimide, and 35.6 mM sodium phosphate monobasic. These micro-columns were then incubated at 37 °C for 15–30 min, after which DPBS was added to the petri dish.

### 4.2. Neuronal Cell Culture

Female Sprague–Dawley rats (Charles River) were the source for primary ventral mesencephalic neurons, a midbrain region previously shown to be enriched in dopaminergic neurons [40]. Carbon dioxide was used to euthanize timed-pregnant rats (embryonic day 14), following which the uterus was extracted. The brains were removed in Hank’s balanced salt solution (HBSS) and the ventral midbrain was isolated [41]. The ventral midbrains were dissociated in accutase for 10 min at 37 °C. The cells were centrifuged at a relative centrifugal force (RCF) of 200 for 5 min and resuspended at 1–2 million cells/mL in standard media consisting of Neurobasal medium + 2% B27 + 1% fetal bovine serum (Atlanta Biologicals) + 2.0 mM L-glutamine + 100 µM ascorbic acid + 4 ng/mL mouse basic fibroblast growth factor (bFGF) + 0.1% penicillin-streptomycin.

Enriched dopaminergic neuron populations were attained utilizing fluorescent-activated cell sorting (FACS) (see Figure 1). Here, transgenic male Sprague–Dawley rats (NTac:SD-Tg(TH-EGFP)24Xen; Taconic model #12141-M) expressing GFP in their dopaminergic neurons were bred with wild–type Sprague-Dawley female rats. Timed-pregnant Sprague–Dawley rats were euthanized via carbon dioxide exposure, the uterus was extracted, and the embryos were isolated (embryonic day 14). The brains were removed in HBSS containing 20 mM glucose and 100 µM ascorbic acid. The ventral midbrains were dissociated in accutase for 10 min at 37 °C. The cells were centrifuged at a RCF of 200 for 5 min and resuspended at 6 million cells/mL in a sorting buffer consisting of HBSS + 20 mM glucose + 100 µM ascorbic acid + 0.1% penicillin-streptomycin + 1.4% bovine serum albumin + 25 mM HEPES. Next, FACS was performed on a BD Influx cell sorter to isolate GFP+ cells. These cells were collected in a buffer containing standard conditioned media + 4% bovine serum albumin + 25 mM HEPES. Following sorting, the collected cells were resuspended at 1–2 million cells/mL in standard conditioned media. Standard conditioned media were generated by placing 12.5 mL of Neurobasal + 1% fetal bovine serum (Atlanta Biologicals) in a T-75 flask containing cortical astrocytes for 24 h. Next, the media was removed from the flask and the following components were added: 2% B27 + 2.0 mM L-glutamine + 100 µM ascorbic acid + 4 ng/mL mouse basic fibroblast growth factor (bFGF) + 0.1% penicillin-streptomycin. High-concentration growth media consisted of Neurobasal medium + 2% B27 + 1% fetal bovine serum (Atlanta Biologicals, Flowery Branch, GA, USA) + 2.0 mM L-glutamine + 100 µM ascorbic acid + 0.1% penicillin-streptomycin + 12 ng/mL mouse bFGF + 10 ng/mL brain-derived neurotrophic factor (BDNF) + 10 ng/mL glial cell-derived neurotropic factor (GDNF) + 10 ng/mL ciliary neurotropic factor (CNTF) + 10 ng/mL cardiotrophin.

Dopaminergic neuron aggregates were created based on protocols previously described [19,42,43,44]. Briefly, custom-built arrays of inverted pyramidal wells were fabricated using polydimethylsiloxane (PDMS) (Sylguard 184, Dow Corning, Midland, MI, USA) cast from a 3D-printed mold and placed in a 12-well plate. Twelve microliters of the dopaminergic cell suspension were transferred to each pyramidal well, and the 12-well plate was centrifuged at 1500 rpm for 5 min, after which 2 mL of standard media was placed on top of each array. The centrifugation resulted in the forced aggregation of neurons (approximately 3200 cells per aggregate). The wells were then incubated overnight. At the time of plating, the DPBS was removed from the dishes containing the micro-columns and replaced with media. Using forceps, the aggregates were inserted into one (unidirectional) or both (bidirectional) ends of the micro-columns, and the cultures were placed in an incubator (see Figure 1).

Female Sprague–Dawley rats (Charles River, Wilmington, MA, USA) were the source of primary striatal neurons. Carbon dioxide was used to euthanize timed-pregnant rats (embryonic day 18), after which the uterus was extracted. To isolate striatal neurons, the brains were removed in HBSS and striata were isolated. The striata were dissociated in trypsin (0.25%) + ethylenediaminetetraacetic acid (EDTA) (1 mM) for 12 min at 37 °C. The trypsin-EDTA was then removed, and the tissue was triturated in HBSS containing DNase I (0.15 mg/mL). The cells were centrifuged at 1000 rpm for 3 min and resuspended at 1–2 million cells/mL in the Neurobasal medium + 2% B27 + 0.4 mM L-glutamine. Striatal aggregates were created and inserted into TE-NSPs as described above for dopaminergic neurons. The striatal aggregates were inserted into the vacant ends of the dopaminergic neuron TE-NSPs between 3 days in vitro (DIV) and 10 DIV.

In some instances, constructs were transduced with an adeno-associated virus (AAV) vector to induce GFP expression (AAV2/1.hSynapsin.EGFP.WPRE.bGH, UPenn Vector Core, ~3.2 × 10^10^ genome copies/mL). Here, at 3 DIV, the TE-NSPs were incubated overnight in media containing the vector, and the cultures were rinsed with media the following day.

### 4.3. Immunocytochemistry

TE-NSPs were fixed in 4% formaldehyde for 35 min and permeabilized using 0.3% Triton X100 plus 4% horse serum for 60 min. Primary antibodies were added (in phosphate-buffered saline (PBS) + 4% serum) at 4 °C for 12 h. The primary antibodies were the following markers: (1) β-tubulin III (1:500, Sigma-Aldrich, St. Louis, MO, USA, cat #T8578), a microtubule element expressed primarily in neurons; (2) tyrosine hydroxylase (TH; 1:500, Abcam, cat #AB113), an enzyme involved in the production of dopamine; (3) microtubule-associated protein 2 (MAP-2) (1:500, Millipore, cat #AB5622), a microtubule-associated protein found in neuronal somata and dendrites; (4) vesicular glutamate transporter 2 (VGLUT2) (1:500, Abcam, cat #AB79157), a protein expressed in glutamatergic neurons; (5) Parvalbumin (1:500, Abcam, cat #AB11427), a protein expressed in GABAergic neurons; (6) glutamic acid decarboxylase 65 (GAD65) (1:500, Abcam, cat #AB49832), a protein expressed in GABAergic neurons; (7) glutamic acid decarboxylase 67 (GAD67) (1:500, Abcam, cat #AB49832), a protein expressed in GABAergic neurons; (8) dopamine-and-cAMP-regulated neuronal phosphoprotein (DARPP-32) (1:250, Abcam, cat #AB40801), a protein found in striatal medium-sized spiny neurons; (9) synapsin 1 (1:1000, Synaptic Systems, cat #106001), a protein expressed in synaptic vesicles of the central nervous system; (10) the dopamine transporter (1:500, Abcam, cat #AB111468), a plasma membrane protein that recycles extrasynaptic dopamine back into the cell; (11) dopamine receptor 1 (1:500, Novus Biologicals, cat #NB110-60017); (12) bassoon (1:500, Synaptic Systems, cat #141011), a presynaptic scaffolding protein; (13) RIM 1 (1:500, Synaptic Systems, cat #140003), a protein in the active zone of neurotransmitter release; and (14) NeuN (1:500, Millipore, cat #ABN91), a neuronal nuclear antigen. Appropriate fluorescent secondary antibodies (Alexa-488, −594, and/or −649 at 1:500 in PBS + 30 nM Hoechst + 4% serum) were added at 18–24 °C for 2 h.

### 4.4. Live/Dead Assay

Calcein AM (Sigma-Aldrich) and ethidium homodimer (Life Technologies, Carlsbad, CA, USA) were used to perform Live/Dead assays on control cultures and TE-NSPs (n = 5 controls, n = 6 TE-NSPs) at 14 DIV. Cultures were rinsed with DPBS, after which they were incubated in a solution of 4 mM calcein AM (labeling the full intracellular compartment of live cells green) and 2 mM ethidium homodimer (labeling the nuclei of dead/dying cells red) for 30 min at 37 °C. Following incubation, the cultures were rinsed three times with DPBS.

### 4.5. In Vitro Microscopy and Data Acquisition

For in vitro analyses, TE-NSPs were imaged using phase contrast and fluorescence on a Nikon Eclipse Ti-S microscope with image acquisition using a QiClick camera interfaced with Nikon Elements. In order to determine the length of neurite penetration, the longest observable neurite in each TE-NSP was measured from the proximal end of the neuronal aggregate after fixation. For in vitro immunocytochemistry analyses, cultures and TE-NSPs were fluorescently imaged using a Nikon A1RSI Laser Scanning Confocal microscope. All TE-NSP confocal reconstructions were from full-thickness z-stacks. In order to determine dopaminergic purity, the number of TH+ neurons was divided by the number of β-tubulin III+ neurons at 14 DIV.

### 4.6. In Vitro Fast Scan Cyclic Voltammetry

At 24–35 DIV, TE-NSPs were transferred to a testing chamber and flushed with culture media. A carbon-fiber electrode (150–200 µm length × 7 µm diameter) was inserted into the dopaminergic aggregate, the dopaminergic axon terminals, or the striatal end target and a bipolar stimulating electrode (Plastics One, Roanoke, VA, USA) was placed across the same location. Dopamine release was elicited using electrical pulse trains (20–30 Hz, 5 ms pulse width, 0.5–1 s, monophasic) every 5–15 min and recorded using Demon Voltammetry and Analysis Software [45]. The potential of the carbon-fiber electrode was linearly scanned from −0.4 to 1.2 V and back to −0.4 V vs. Ag/AgCl. A voltammeter/amperometer (Chem-Clamp; Dagan Corporation, Minneapolis, MN, USA) was used to scan at a rate of 400 V/s, and cyclic voltammograms were recorded every 100 ms. Electrode calibrations using known dopamine concentrations (1–10 μM) were used to calculate the concentration of electrically evoked dopamine release at the peak oxidation potential for dopamine in consecutive voltammograms. For the TE-NSPs treated with the dopamine transporter (DAT) inhibitor GBR12909, increasing concentrations of GBR12909 were perfused through the testing chamber. Concentrations of GBR12909 included 0.0 µM, 0.02 µM, 0.2 µM, 2.0 µM, and 20 µM. Dopaminergic neurons were incubated in each concentration for 30 min before the evoked dopamine release was recorded.

### 4.7. Sheath Fabrication

Protective “sheaths” were fabricated to prevent the implantation needle from coring out the brain and damaging the TE-NSPs during insertion into the brain. The sheaths were generated using an electrical bag sealer to seal the end of a 2 cm piece of tubing (Microspec, Isoplast 2510, ID: 559 µm, OD: 686 µm). The seal created by the bag sealer was then cut to a length of 0.5 mm, and three cuts parallel to the tubing were also created through the seal. These cuts enabled the escape of air when the sheath was slid over the insertion needle. The cuts also weakened the seal, which allowed the seal to be ripped following the insertion of the implantation needle into the brain.

### 4.8. Transplantation of TE-NSPs

Male athymic rats lesioned via the neurotoxin 6-hydroxydopamine (injected into the SNpc) were procured from Charles River (n = 51 rats). These rats (205–270 g) were anesthetized with isoflurane and mounted in a stereotactic frame. The scalp was cleaned with betadine, bupivacaine was injected along the incision line, and a midline incision was made to expose Bregma. A 5 mm craniectomy was centered at the following coordinates in relation to Bregma: +5 mm (AP), 2.1 mm (ML). A TE-NSP (21 DIV) was loaded into a needle (OD: 534 µm, ID: 420 µm; Vita Needle, Needham, MA, USA), a protective sheath was slid over the needle, and the needle was attached to a Hamilton syringe mounted on a stereotactic arm. The stereotactic arm was positioned at 38° relative to the horizontal plane, the dura was opened, and the needle was lowered into the brain to a depth of 12.0 mm. The needle was rigidly affixed in place by the stereotactic equipment. A pair of forceps was used to grab the exposed section of the sheath and pull the sheath upwards; this action ripped through the seal on the end of the needle and exposed the TE-NSP within the brain. A stationary arm was positioned to make contact with the plunger of the Hamilton syringe and hold the plunger in place as the needle was withdrawn from the brain. This implantation method “laid out” the TE-NSP within the brain, rather than expelling it from the needle. The scalp was sutured and closed and buprenorphine was provided for postoperative analgesia. Acellular micro-columns and micro-columns containing aggregates (utilized at 1–3 DIV) were transplanted following the same procedure. Solitary aggregates (21 DIV) were transplanted using the same procedure with a different needle (OD: 534 µm, ID: 152 µm). Aggregates transplanted into the striatum were lowered to a depth of 5 mm within the brain, rather than 12 mm. Animals survived for 15 min (TE-NSPs only, n = 6), 1 week (see groups below), or 1 month (see groups below). For the 1-week time points, experimental groups were (i) fully grown, enriched TE-NSPs (n = 3 rats), (ii) aggregates only delivered into the striatum (n = 2), and (iii) aggregates only delivered into SN (n = 2). For the 1-month time point, experimental groups were (i) fully grown, enriched TE-NSPs (n = 20), (ii) aggregates within micro-columns (n = 3), (iii) acellular micro-columns containing collagen only (n = 6), (iv) fully grown, impure TE-NSPs (n = 3), and (v) lesioned, non-repaired animals (n = 6). At the 1-week time point, all animals were examined via histology, whereas at the 1-month time point, animals were used for either voltammetry or histology.

### 4.9. Immunohistochemistry

Animals were anesthetized and then euthanized via transcardial perfusion with heparinized saline followed by 10% formalin. Brains were removed, placed in 4% paraformaldehyde for 24 h, and then prepared for tissue clearing or cryosectioning. For tissue clearing, a combination of the Visikol Inc. (Union Township, NJ, USA) histology protocol [46] and the iDISCO protocol [47] was followed. Briefly, brains were cut into 2 mm sections using a vibratome, dehydrated in a series of methanol washes, incubated in dichloromethane (66%), treated with hydrogen peroxide, and then rehydrated. Sections were permeabilized in a buffer containing PBS, 0.2% Triton X-100, 0.3 M glycine, and 20% DMSO for 2 days. Sections were blocked with 6% horse serum for 2 days and then incubated in the following primary antibodies for 7 days: (1) Sheep anti-TH (1:100, Abcam, cat #AB113); (2) chicken anti-GFP (1:100, Aves Labs, cat #GFP-1020); and (3) mouse anti-synapsin (1:100, Synaptic Systems, cat #106001). Next, sections were incubated in secondary antibodies (1:250) for 7 days. Sections were then dehydrated, treated with Visikol^®^ HISTO-1^TM^ for 18 h, and imaged in Visikol^®^ HISTO-2^TM^.

For cryosectioning, brains were blocked sagittally, put into 30% sucrose until saturated, and then frozen. Sections were cryosectioned at 35 µm, mounted on slides, and processed for immunohistochemistry. Frozen sections were blocked with 5% normal horse serum in 0.1% Triton-x/PBS for 30–45 min. Primary antibodies (Rabbit anti-TH, 1:750, Abcam, #AB112; Mouse anti-Tuj1, 1:1000, Sigma, #T8578) were applied to the sections in a 2% horse serum/Optimax buffer for 2 h at room temperature. Secondary antibodies (1:1000) were applied in 2% horse serum/PBS for 1 h at room temperature. Sections were counterstained with Hoechst.

### 4.10. Microscopy and Data Acquisition from In Vivo Tissue

For the analysis of TE-NSPs post-transplant, TE-NSPs were fluorescently imaged using either a Nikon A1RSI Laser Scanning Confocal microscope or a Nikon A1R HD Multiphoton microscope. A skilled researcher blinded to the enrollment group made all assessments. Each section was analyzed to assess the presence, architecture, and outgrowth/integration of TE-NSP neurons/neurites. Within the histologically assessed samples, the health of the implanted aggregates or constructs was described as exhibiting a clear presence of cell survival or no evidence of cell survival. Implanted aggregates or constructs were also classified based on presence or absence of axonal tracts spanning the length of the micro-column. These binary outcome metrics were utilized to determine the frequency of cell survival or axonal growth across implant conditions at early and later time points.

### 4.11. Fast Scan Cyclic Voltammetry in Brain Slices

At the time of sacrifice, carbon dioxide followed by decapitation was used to euthanize the animals. Brains were removed and placed in ice-cold artificial cerebrospinal fluid (aCSF) containing, in mM, NaCl (126), KCl (2.5), NaH2PO4(1.2), CaCl2(2.4), MgCl2(1.2), NaHCO3(25), glucose (11), and l-ascorbic acid (0.4), with pH adjusted to 7.4 [48]. A vibratome was used to cut the brain within the sagittal plane. At the depth containing the transplanted TE-NSP (or micro-column, aggregate, etc.), a 400 µm thick brain slice encapsulating the TE-NSP was taken. The slice was transferred to a bath where it was perfused with oxygenated aCSF at room temperature for 1 h prior to recording studies. Following this incubation period, the slice was transferred to a testing chamber at 32 °C, where it was continuously perfused with oxygenated aCSF. A carbon-fiber electrode (150–200 µm length × 7 µm diameter) was inserted into the striatum and a bipolar stimulating electrode (Plastics One, Roanoke, VA, USA ) was placed across the same location. Dopamine release was elicited using electrical pulse trains (20–30 Hz, 5 ms pulse width, 0.5–1 s, monophasic) every 5–15 min and recorded using Demon Voltammetry and Analysis Software [45]. The potential of the carbon-fiber electrode was linearly scanned from −0.4 to 1.2 V and back to −0.4 V vs. Ag/AgCl. A voltammeter/amperometer (Chem-Clamp; Dagan Corporation) was used to scan at a rate of 400 V/s, and cyclic voltammograms were recorded every 100 ms. Electrode calibrations using known concentrations of dopamine (1–10 μM) were used to calculate the concentration of the electrically evoked dopamine release at the peak oxidation potential for dopamine in consecutive voltammograms.

### 4.12. Statistical Analyses

The normality of all data was examined, and for normal data distributions, one-way ANOVAs were performed; when differences existed between groups, post-hoc Tukey’s pair-wise comparisons were performed. For non-normal data, unpaired, non-parametric, two-sided Mann–Whitney tests were employed to determine if there were statistically significant differences between groups. Binomial data from the in vivo histological assessment were utilized to determine whether the observed neuronal aggregate survival or axonal growth significantly differed from the expected survival or growth, respectively (with each compared to the performance of implanted aggregates alone or within micro-columns at the matching time point). Two-tailed binomial tests were completed in Prism version 9.4.1, GraphPad Software Inc. San Diego, CA, USA. For all statistical tests, *p* < 0.05 was required for significance.

## 5. Conclusions

The stereotypical neurodegeneration that occurs in PD deprives the striatum of crucial dopaminergic inputs and thereby interrupts important motor feedback pathways. TE-NSPs are designed to be the first strategy capable of replacing the structure and function of the lost nigrostriatal pathway and restoring dopamine tone in the striatum under endogenous regulation. In the current work, TE-NSPs were generated using GFP+ dopaminergic neurons that projected long axonal tracts through a biomaterial encasement and were capable of approximating the nigrostriatal pathway in rats. When implanted into a rat model of PD-like neurodegeneration, TE-NSPs were found to improve dopamine levels in the striatum while demonstrating improved neuronal survival and axonal architecture in comparison to alternative implant paradigms, including more common purely cell-based strategies. Given these promising results, future work should optimize TE-NSP bio-fabrication using a translatable human cell source and improve both the potency and consistency by refinement of the “dose” (e.g., the number of neurons and density of axons) and timing (e.g., maturation) of the implant, or other potentially important parameters. Such next-generation constructs may provide a transformative and scalable solution to directly replace neurons in the SNpc, restore axon-mediated dopaminergic signaling in the striatum, and thereby alleviate the cause of motor symptoms in PD. In the coming era of restorative neurosurgery, TE-NSPs have the potential to enhance PD treatment and improve outcomes for patients worldwide.

## Data Availability

The data presented in this study are available upon request to the corresponding author.
